# London Lancet

**Published:** 1844-05

**Authors:** 


### LONDON LANCET.

The publication of this medical newspaper in this country was suspended for some time, but we are glad to have in our power to announce that it has been again resumed. Our readers will be pleased to hear it. The Lancet is one of the most spirited and racy publications of the day, and ought to have a wide circulation. Its present publisher is Thomas Walsh, 162, Nassau street, New York.

